# A Pilot Study of the Diagnosis of Oral Cancer Through the Development of an AI Application

**DOI:** 10.3390/dj14070429

**Published:** 2026-07-12

**Authors:** Vasileios Zisis, Pavlos Theodosiadis, Christina Charisi, Konstantinos Poulopoulos, Petros Papadopoulos, Evangelos Parcharidis, Georgios Parlitsis, Effimia Stergiadou, Chrysomallis Dimitris, Athanasios Poulopoulos

**Affiliations:** 1 Department of Oral Medicine/Pathology, School of Dentistry, Aristotle University of Thessaloniki, 54124 Thessaloniki, Greece; pavlos.theodosiadis@gmail.com (P.T.); akpoul@dent.auth.gr (A.P.); 2 Department of Dentistry (Oral Medicine-Oral Pathology), School of Dentistry, European University, Diogenous Street 6, Nicosia 2404, Cyprus; 3 Department of Dentoalveolar Surgery, Surgical Implantology and Radiology, School of Dentistry, Aristotle University of Thessaloniki, 54124 Thessaloniki, Greece; 4 Department of Mechanical Engineering, Eindhoven University of Technology, Atlas Building, De Zaale, 5612 AZ Eindhoven, The Netherlands; 5 DeepNexus S.A., 67131 Xanthi, Greece

**Keywords:** artificial intelligence, oral medicine, dentistry, oral cancer, oral squamous cell carcinoma, OSCC

## Abstract

**Background/Objectives**: Artificial intelligence (AI) has emerged as a transformative tool in oral medicine, where it holds significant promise for enhancing the diagnosis, treatment, and management of oral cancer. Our team aimed to develop a new tool, capable of diagnosing oral cancer utilizing the capabilities of AI. **Methods**: The task we aim to solve from computer vision’s perspective is an object detection task. Under this context, a detection is essentially a bounding box drawn around an oral lesion accompanied by the disease’s description. To solve the task at hand, we collected and annotated a wide set of images which were used for training our model. Specifically, we used 205 images of Oral Squamous Cell Carcinoma (OSCC). Following common practice, 80% of the total images were allocated for training, 10% for validation, and 10% for testing. The training set was used to optimize the model’s parameters across multiple iterations. The validation set served to prevent overfitting during training and to guide hyperparameter tuning. Lastly, the test set was used for evaluation of data that had not been previously seen by the model and had not influenced any decisions regarding its architecture or hyperparameters. Moreover, during evaluation, we supplemented the test set by adding 100 images of healthy mucosa to examine whether the model generated false positives on healthy tissue. To broaden the dataset’s coverage, we generated synthetic images by applying data augmentation techniques such as random rotation, scale and noise injection. The model’s architecture was based on YOLO11, which is a widely spread neural network architecture known for its balance between efficiency and performance, used in object detection tasks. **Results**: The model’s detections were accompanied by a confidence measure, which was used to filter out those with low confidence, and one could choose a lower threshold for maximizing precision or a higher threshold for maximizing recall. Among images that correspond to oral cancer (oral squamous cell carcinoma), the model achieved 59% precision and 41% recall on the validation set and 56% precision and 42% recall on the test set. The limitations of this study include the single institutional design and the relatively small sample size. The number of images used for model training was relatively small (205 images of oral squamous cell carcinoma), which may limit the generalizability of the findings. The main limitation is that the model distinguishes oral squamous cell carcinoma from healthy mucosa. In routine clinical practice, however, the diagnostic challenge is to differentiate oral cancer from a variety of benign and potentially malignant disorders that may present with similar clinical features. The inclusion of other oral lesions is planned in future studies. **Conclusions**: The efficiency of our AI application may be considered as encouraging, taking the pilot nature of the study into consideration. More clinical photos and better training of the model may lead to better precision and recall, enabling its inclusion in standard clinical practice. Larger multicenter datasets will be required for clinical implementation. AI-driven tools can assist in risk stratification, helping clinicians determine the best treatment plans by analyzing patient data and predicting the likelihood of recurrence or metastasis. AI’s potential extends beyond diagnosis and treatment; it also contributes to monitoring patient outcomes. As research and technology evolve, AI’s role in oral cancer is likely to become increasingly indispensable.

## 1. Introduction

Artificial intelligence (AI) is defined as the sum of capabilities of computers and technological devices to copy human intelligence and execute human tasks [1]. Problem solving, learning, decision-making, and comprehending language constitute such tasks. The precise mechanism involves neural networks (NNs) utilizing artificial neurons that are analogous to those of humans and can perform these tasks [2]. AI was initially described in the 20th century and is currently evolving from just a theoretical background to various applications affecting the whole spectrum of human activity, including healthcare [1,3]. Healthcare by far exceeds other AI applications with regard to equity investment [3]. The necessary algorithms, when provided with sufficient datasets, identify patterns and make predictions or decisions based on those patterns [4]. Machine learning and deep learning allowed for the development of such algorithms, thus creating systems able to enhance their outcomes timewise by expanding the datasets [5]. In theory, AI may identify patterns incomprehensible to humans; therefore, AI is suitable for analyzing specifically complicated information and datasets. The main outcome is accurate analytics in real-time decision-making even in dynamic environments [3,4]. The diagnostic imaging industry, specifically oral and maxillofacial radiology in terms of dental science, is already accustomed to the implementation of AI tools, since they simplify routine checkup examinations, lower the cost by automating the process, elevate the treatment quality by sustaining a state-of-the-art protocol, and erase possible human errors. Academic teaching is also affected by developing individualized teaching which corresponds to the needs of each student [3]. With AI developing at such a rapid pace, there is a chance that it may change healthcare as we know it by being applied in clinical practice. In order to ensure that healthcare practitioners have the information they need to effectively incorporate AI into patient care, it is crucial to record and share data about AI’s function in clinical practice. Nevertheless, it is still under investigation in the international scientific literature, considering how exactly and to what extent AI may assist in the detection, prevention, and treatment of dental problems [5]. The same applies to the field of oral medicine where, so far, a breakthrough in predictive diagnostics is still missing.

AI performs better than conventional methods for oral cancer screening, analysis, and prediction [6]. Convolutional neural networks can be used to diagnose mouth cancer from medical images. In places with limited resources, screening is accessible and inexpensive thanks to smartphones and AI-enabled telemedicine. AI techniques use patient data to forecast the risk of oral cancer [6]. AI can also handle data heterogeneity, limited longitudinal research, clinical practice inclusion, ethical and legal challenges, and treatment planning utilizing histopathology pictures. Uniform standards, long-term research, ethical and legal frameworks, and training for healthcare professionals are all potential future developments [6]. The future of AI in oral cancer diagnosis hinges on enhancing algorithm accuracy and usability, as well as adapting it to varied clinical scenarios [7]. To eliminate prejudice, the focus should be on improving AI algorithms, particularly keeping them equal and prepared across many diverse datasets [7]. The integration of AI techniques in routine dental check-ups may be an important stage of early detection.

A recent systematic review was conducted including five databases (PubMed, Embase, Cochrane Library, Web of Science, and Scopus) and 63 studies that analyzed data input modalities and the evolution of AI algorithms [8]. AI models showed a tendency toward multimodal fusion, lightweight design, high-performance development, and excellent sensitivity and specificity in identifying early oral lesions and discriminating precancerous lesions [8]. Nevertheless, the majority of research encountered difficulties such as inadequate sample sizes, little external validation, and poor model interpretability [8].

The aim of this research project was to determine how an AI tool may be utilized in practice, in order to correctly diagnose conditions in the field of Oral Medicine and, in particular, oral cancer.

## 2. Material and Method

The aim of the project “Diagnosis of Oral Diseases, using Artificial Intelligence” is the treatment and prognosis of diseases of the oral cavity, using Artificial Intelligence (AI) techniques. For this purpose, a “smart” Information System (smart-IS) is implemented, which is addressed to doctors, dentists and hospital institutions. The smart-IS serves as a Computer-Aided Diagnosis (CAD) tool, allowing clinicians to capture intraoral images of suspicious lesions and get a preliminary diagnosis for those lesions. At the core of the system, there is a deep neural network architecture trained on a large collection of annotated images provided by the Oral Medicine and Oral Pathology scientific group at Aristotle University of Thessaloniki. Having such a tool at hand could provide huge and important benefits to the field, contributing dynamically to both the early prognosis and treatment of possible diseases, as well as to the saving of resources and time, important factors that are often lacking currently in health systems.

The project team consists of two groups. The first is the Oral Medicine and Oral Pathology scientific group of the Dentistry Department at Aristotle University of Thessaloniki, and the second is DeepNexus S.A. The university team collected, organized, and annotated the data through the smart-IS platform. It also performed independent testing of the integrated CAD system. DeepNexus S.A. developed and implemented the smart-IS, which includes an appropriate deep learning architecture for the detection and classification of oral lesions. The project lasted 13.5 months and was funded by the National Recovery and Resilience Plan ‘Greece 2.0’ under NextGenerationEU. In addition, the project received approval from the Research Ethics and Deontology Committee of the Aristotle University of Thessaloniki (REDC AUTH) with identification number 118478/2025 on 7 May 2025.

The task we aim to solve from computer vision’s perspective is an object detection task. Under this context, a detection is essentially a bounding box drawn around an oral lesion accompanied by the disease’s description. To solve the task at hand, we collected and annotated a wide set of images which were used for training our model. Specifically, we used 205 images of Oral Squamous Cell Carcinoma (OSCC). Following common practice, 80% of the total images were allocated for training, 10% for validation, and 10% for testing. The training set was used to optimize the model’s parameters across multiple iterations. The validation set served to prevent overfitting during training and to guide hyperparameter tuning. Lastly, the test set was used for evaluation of data that had not been previously seen by the model and had not influenced any decisions regarding its architecture or hyperparameters. Moreover, during evaluation, we supplemented the test set by adding 100 images of healthy mucosa to examine whether the model generated false positives on healthy tissue. To broaden the dataset’s coverage, we generated synthetic images by applying data augmentation techniques such as random rotation, scale and noise injection. The model’s architecture is based on YOLO11, which is a widely spread neural network architecture known for its balance between efficiency and performance.

All the cases were histopathologically confirmed prior to the annotation. The anonymization of data was conducted as per rules established by the Institutional Ethics Committee.

The annotation process of each photo was conducted by an oral medicine specialist and then it was further validated and approved by the rest of the group of oral medicine practitioners, all highly specialized, including MSc students, PhD students and PhD graduates (V.Z., C.C., K.P., P.P., E.P., E.S., A.P.) led by Professor A.P., the principal investigator (P.I.) of the study.

Given the set of clean and annotated images, data augmentation methods were applied to increase the set’s diversity. These methods generate new synthetic images by applying transformations to the original ones. Exposing the model to the same objects (e.g., oral lesions) under various conditions improves its overall detection performance. More importantly, it enhances the model’s generalization capability, which refers to preserving effectiveness across different settings while applied to the same task. Specifically, we use the following data augmentation strategies:

1. Hue Saturation Value (HSV) color space modification (H: 0.015, S: 0.7, V: 0.4),
2. Horizontal flipping (*p* = 0.5),
3. Random scaling (range = 0.5),
4. Random translation (range = 0.1),
5. Mosaic generation (*p* = 0.5), and
6. Random erasing (*p* = 0.4).

These transformations affect an image’s colors or brightness and modify the lesion’s depiction in such a way that takes into account data symmetries. For example, a given lesion may appear on the left or right side, under better or worse lighting conditions, with the camera closer or further away from it.

In order to imitate the clinical conditions of a typical clinical practice, we took photos with a variety of equipment; cameras and smartphones were equally used, both older and more modern devices. Furthermore, our study was both retrospective and prospective in nature. We used clinical images from the archives of our department, as well as clinical images of patients who visited our clinic after the commencement of the project.

## 3. Results

To evaluate our method’s performance, we use two standard metrics for object detection tasks: precision and recall. In essence, precision quantifies the proportion of the correct positive predictions with respect to the total amount of positive predictions (i.e., the chance that the model’s detection corresponds to an actual lesion) and recall/sensitivity quantifies the proportion of the correct positive predictions with respect to the total amount of annotated oral lesions (i.e., the chance of finding a lesion if such a lesion exists on the image). Given that the model’s detections are accompanied by a confidence measure, which is used to filter out those with low confidence, one can choose a lower threshold for maximizing precision or a higher threshold for maximizing recall. This is known as the precision–recall trade-off.

**Figure 1:**
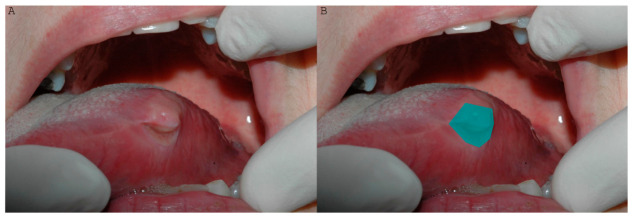
(**A**) Oral squamous cell carcinoma on the left lateral border of the tongue. (**B**) The blue field covers the lesion, and its periphery is annotated by an oral medicine specialist.

**Figure 2:**
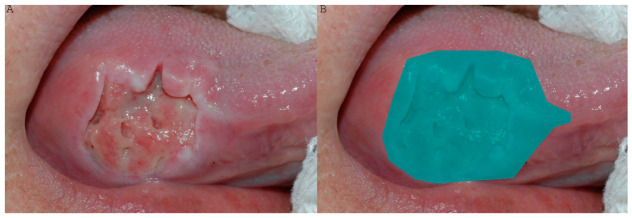
(**A**) Oral squamous cell carcinoma on the right lateral border of the tongue. (**B**) The blue field covers the lesion, and its periphery is annotated by an oral medicine specialist.

**Figure 3:**
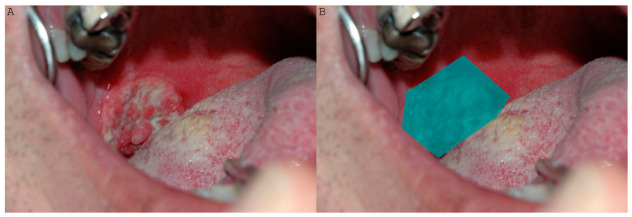
(**A**) Oral squamous cell carcinoma on the right half of the soft palate. (**B**) The blue field covers the lesion, and its periphery is annotated by an oral medicine specialist.

**Figure 4:**
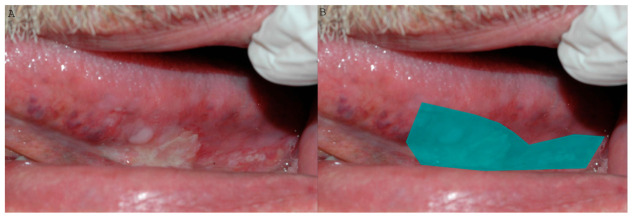
(**A**) Oral squamous cell carcinoma on the left lateral border of the tongue. (**B**) The blue field covers the lesion, and its periphery is annotated by an oral medicine specialist.

**Figure 5:**
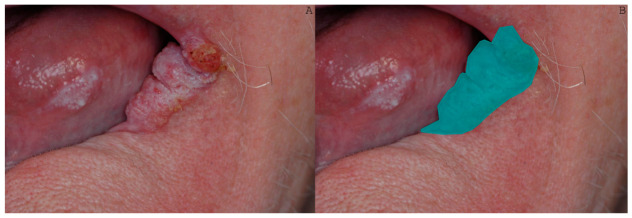
(**A**) Oral squamous cell carcinoma on the left corner of the mouth. (**B**) The blue field covers the lesion, and its periphery is annotated by an oral medicine specialist.

The annotation process may be witnessed in the following Figure 1, Figure 2, Figure 3, Figure 4 and Figure 5.

We chose the threshold in such a way that balances the two metrics by maximizing the model’s F1 score on the validation set. F1 score is defined as the harmonic mean of the precision and recall:

$$f1=\frac{2\cdot precision\cdot recall}{precision+recall}.$$

Among images that correspond to oral cancer (oral squamous cell carcinoma), the model achieves 59% precision and 41% recall on the validation set and 56% precision and 42% recall on the test set. Our study was both retrospective and prospective in nature. We used clinical images from the archives of our department, as well as images recently photographed in our clinic with modern cameras and smartphones.

**Figure 6:**
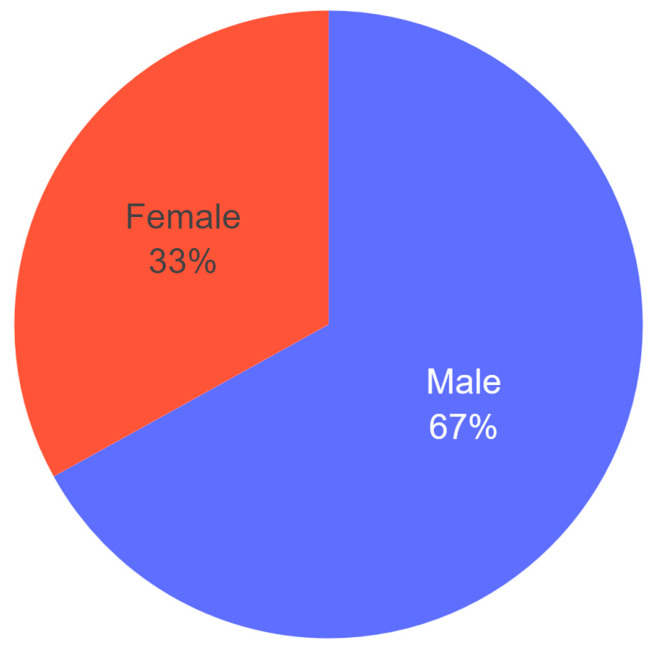
Distribution of the patients’ gender.

**Figure 7:**
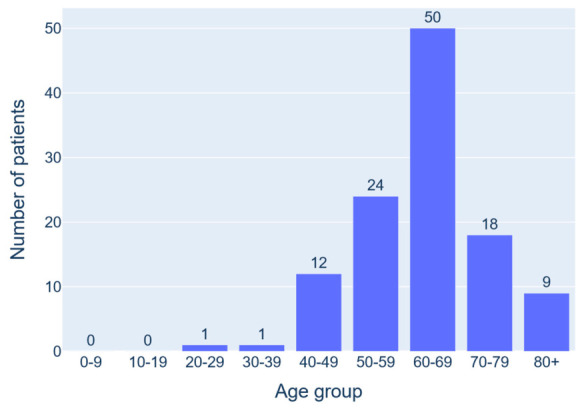
Distribution of the patients’ age.

**Figure 8:**
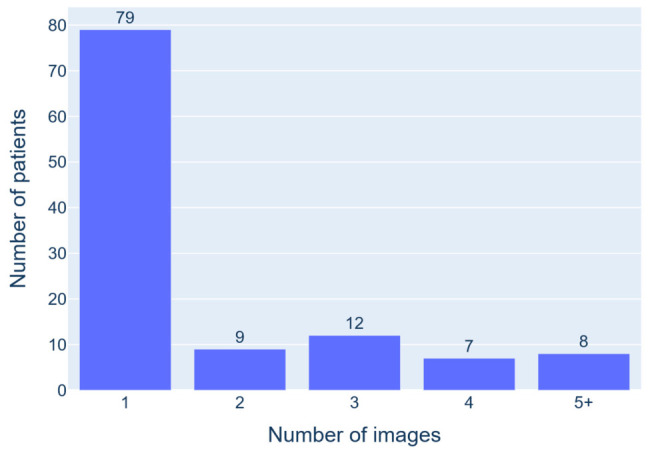
Distribution of the number of images per patient.

The results suggest that image-based medical diagnosis of specific oral lesions using neural network architectures is possible and motivate further exploration. Future iterations will incorporate more sophisticated methodologies together with more image samples to transition towards adoption in the clinical environment. A pie chart of the gender distribution (Figure 6) and an illustration of the age distribution (Figure 7) are shown, as well as the number of images per patient (Figure 8), the descriptive statistics of the patient’s age (Table 1), and the number of images per patient (Table 2).

## 4. Discussion

AI allows for the development of tools which may solve problems similarly to human intelligence [9]. These tools are classified based on their capabilities, properties, and way of learning and analyzing datasets. In theory, AI may be perceived as weak, capable of dealing with very specific tasks, medium, equivalent to a human brain, or super, surpassing the human brain [10].

Machine learning (ML), a branch of artificial intelligence, enables computers to improve their performance over time by learning from data without explicit programming for specific tasks [10]. ML algorithms use statistical methods and historical data to identify patterns, make decisions, and predict outcomes. Convolutional neural networks (CNNs), a form of deep learning (DL), are particularly effective for processing complex and large images by extracting features through layered filters [10]. Deep learning (DL) can be described as an advanced form of machine learning (ML). It uses deep neural networks, which consist of many hidden layers that process data in a structured manner [10]. This allows machines to learn complex patterns, form connections, and optimize inputs by assigning different weights, making DL particularly powerful within the field of ML.

The shortage of dental and oral healthcare professionals is putting additional pressure on already strained health systems and threatening the affordability and accessibility of care, especially as the burden of oral diseases is expected to rise due to demographic and epidemiological changes [11]. Many see digital technologies, such as artificial intelligence (AI), as a way to enhance decision-making and improve process efficiency. In oral and dental care, AI offers significant potential to improve treatment quality for more patients by increasing efficiency, safety, and effectiveness [12].

Oral and maxillofacial medicine, like other medical specialties, presents several ethical and legal issues regarding the application of AI. Artificial intelligence systems used in OMF pathology depend heavily on patient data, particularly those that interpret diagnostic pictures [13,14,15]. One example of privacy laws that must be adhered to while gathering, keeping, and utilizing this kind of data is the General Data Protection Regulation (GDPR) of the European Union [10]. Strict measures are also required for data security and the avoidance of unwanted access. Patients should be informed of the advantages and disadvantages of artificial intelligence (AI) in healthcare. Informed consent becomes even more important when artificial intelligence models are used for diagnostic purposes [16,17].

AI systems have the potential to reinforce any biases found in the datasets used to train them. One typical drawback of AI systems is that, because of their “black box” nature, doctors are frequently unable to understand them. This might be an issue in a clinical setting when understanding the reasoning behind a diagnosis or prognosis is crucial. To ensure the safety and effectiveness of AI systems used in oral and maxillofacial medicine, extensive testing is required. To validate the results of investigations, more randomized control trials are needed like the one from Al Sarem et al. [18]. With a segmentation accuracy of 93.3% and a classification accuracy of 89% for missing tooth areas, the DenseNet169 model demonstrated strong performance in the various phases of CBCT-based detection and classification. Therefore, using this model might be a potential time-saving technique that helps dental implantologists take a big step toward automated dental implant design. Despite such claims, AI systems will have to be validated through established criteria by regulatory agencies.

Accurately identifying and categorizing various maxillofacial cysts and tumors is a challenge for doctors. Future clinical applications of AI for the automated diagnosis of certain diseases show promise. Researchers are focusing on developing artificial intelligence models that are trained using 2D/3D images in order to more accurately classify maxillofacial lesions and cancers [10,19]. Yilmaz et al. showed that by using a forward feature selection approach and a ten-fold cross validation method, the Support Vector Machine (SVM) classifier produced the best classification performance, with 100% accuracy and 100% F-score (F1) [19]. In studies using a split sample validation approach with a forward feature selection algorithm, SVM produced the best classification performance, with 96% accuracy and 96% F1 score. Additionally, SVM produced the greatest results with 94% accuracy and 93.88% F1 score when a forward feature selection approach was used with the leave-one-out (LOOCV) method [19]. A similar study to ours, detecting oral cancer lesions from intra oral patient images, from 2024, reported that the F1, sensitivity and precision results of the artificial intelligence model obtained using the YOLOv5 architecture were found to be 0.667, 0.667 and 0.667, respectively [20].

However, it should be noted that there is a main difference between oral radiology and oral medicine: oral radiology includes lesions with a very typical pathognomonic appearance in the CBCT examination, allowing for such a high-performance rate. Oral medicine, on the other hand, includes lesions with varied clinical appearance, rendering the AI training process much more challenging.

It is currently difficult to develop a fully automated model that can identify tumors and cysts, and early lesion diagnosis still requires human input. In a thorough review including thirteen researchers, Santer et al. found that AI shows potential in detecting suspicious lymph nodes in patients with locally progressed head and neck squamous cell carcinoma. The authors of the study demonstrated that AI could identify lymph nodes with an average accuracy of 86% [21]. Furthermore, the authors claimed that the main limitations of their study were the single institutional design and the small sample size.

Oral cancer is one of the most prevalent malignancies and has a high death rate, making it a significant public health problem. Oral cancer diagnosis and recurrence prediction are the most challenging aspects of AI, as it requires complex data on etiology and risk factors [10,22].

AI has definite benefits over other techniques for detecting oral cancer. This innovation can always learn more due to its versatility. AI calculations can improve prediction power by incorporating new patient data, which reduces treatment costs and eases patients’ financial difficulties. AI learning algorithms trained on cytology, fluorescence, CT, and depth of invasion imaging data can be used to diagnose OC quickly and accurately.

In one study, Sunny et al. employed telecytology (TC), which is the digitalization of cytology slides, to detect OC early [23]. The model showed improved accuracy of 73% in recognizing potentially malignant lesions and 93% in diagnosing malignancies. In order to identify OC and characterize the course of oral cancer, Jeyaraj et al. used a regression-based deep learning technique. When examined on the same photos as the traditional methods, the researchers found that the regression-based approach was 91.4% sensitive in identifying malignant lesions [24]. In terms of diagnostic accuracy, the suggested algorithm model fared better than the gold standard.

Uthoff et al. were able to differentiate between precancerous and malignant lesions in white light and autofluorescence images using convolutional neural networks (CNNs) [25]. However, a major drawback of their study is the small sample size (99 cases in total, six of which were OSCCs). CNNs diagnosed malignant and precancerous tumors more accurately than specialists. AI systems may go through several data sources, assess risk, and suggest specialists in order to diagnose cancer. Research on cancerous tumors, premalignant lesions, and lymph nodes has produced encouraging results for the application of AI in diagnosis and prognosis. These programs may reduce mortality rates if they are effective in promoting early diagnosis and curative treatments.

From the standpoint of our pilot study, we aim to enhance the predictability of our model by increasing the number of photos for training purposes. This purposeful lack of a specific photographing protocol constitutes a main advantage of this study; any AI application with the aim of everyday implementation should be able to handle the variability of technical mediums. On the other hand, this variability is probably the causative factor for the reported percentages of precision and recall. Technicalities in photography (aperture, shutter speed, ISO, focus, and light management) varied due to the different equipment. The skills of different photographers (which include understanding depth of field, white balance, focal length and metering modes) vary as well. Finally, OSCC localization matters. When an OSCC emerges in the posterior third of the oral cavity, involving anatomic areas such as the floor of the mouth, the tongue, the palate and the retromolar triangle, it is very difficult to accurately depict it. The situation worsens further when the patient has pharyngeal reflex.

The limitations of this study include the single institutional design and the relatively small sample size. The number of images used for model training was relatively small (184 images of oral squamous cell carcinoma), which may limit the generalizability of the findings. The main limitation is that the model distinguishes oral squamous cell carcinoma from healthy mucosa. In routine clinical practice, however, the diagnostic challenge is to differentiate oral cancer from a variety of benign and potentially malignant disorders that may present with similar clinical features. The inclusion of other oral lesions is planned in future studies.

## 5. Conclusions

The efficiency of our AI application may be considered as encouraging, taking the pilot nature of the study into consideration. More clinical photos and better training of the model may lead to better precision and recall, enabling its inclusion in standard clinical practice. Larger multicenter datasets will be required for clinical implementation. Together, neural networks, machine learning, and deep learning enable AI systems to learn and improve over time, creating a plethora of opportunities for technological growth. We are still in the early phases of fully utilizing AI for medical diagnosis. However, more details on using AI to identify different oral conditions are becoming available. Artificial intelligence is growing in the dental sector as a result of technical advancements and the digitization of dentistry. Computers may now offer second viewpoints in a number of dentistry disciplines. The use of AI in oral medicine has the potential to significantly increase diagnostic efficiency, speed, and accuracy.

## Figures and Tables

**Table 1:** Descriptive statistics of the patients’ age.

Statistic	Value
Mean	62.51
Std. deviation	11.22
Minimum	21.00
25th percentile (Q1)	56.50
Median (Q2)	63.00
75th percentile (Q3)	69.00
Maximum	85.00

**Table 2:** Descriptive statistics of the number of images per patient.

Statistic	Value
Mean	1.78
Std. deviation	1.38
Minimum	1.00
25th percentile (Q1)	1.00
Median (Q2)	1.00
75th percentile (Q3)	2.00
Maximum	7.00

## Data Availability

The original contributions presented in the study are included in the article. Further inquiries can be directed to the corresponding author.
